# Bone infections: local delivery of antibiotics and their effectiveness.

**DOI:** 10.1097/OI9.0000000000000103

**Published:** 2021-06-15

**Authors:** Peter V. Giannoudis, Michael J. Gardner

**Affiliations:** a Academic Department of Trauma and Orthopaedics, LGI, University of Leeds; b NIHR Leeds Biomedical Research Center, Chapel Allerton Hospital, Leeds, UK; c Department of Orthopaedic Surgery, Stanford University School of Medicine, Palo Alto, CA

Bone infection continues to be the most devastating complication post fracture fixation.^[1]^ Its incidence has been reported to be as high as 5%, although after open fractures can be even higher.^[2]^ Classification systems of the spread of the infection, diagnostic investigations, surgical techniques, and algorithms of treatment have evolved over time aiming to improve outcomes. A multidisciplinary team approach is considered nowadays essential for a timely and personalized strategy to achieve a successful eradication of infection.

However, treatment can be prolonged and not infrequently with unpredictable outcomes, leading to limb loss and psychological sequalae. The socioeconomic impact on the patient, the family, and the health care system can be immense.^[3]^

An important adjunct to surgical debridement for the creation of an aseptic environment in the previous infected anatomical bone region is the administration of pathogen-specific antibiotics. They can be delivered either systemically or locally with the use of cement spacers, cement beads, matrices, scaffolds, continuous irrigation systems, and coated nails.^[4]^

Each of these options of delivery has advantages and disadvantages. Important parameters for the surgeon to consider in terms of the selection process include: the elution characteristics of the carrier, the presence or absence of resistance, the bone penetration profile of the antibiotic, the frequency and severity of side effects related to the antibiotic treatment, the required time length of administration, and the presence of patient comorbidities amongst others.

In this special issue this important topic of local antibiotic administration for the management of bone infection is covered. The material included is by no means comprehensive to cover all the important issues related to the strategies of bone infection eradication. However, the topics included are carefully selected with the aim of increasing the knowledge and awareness of clinicians, highlighting important aspects of their use in the clinical setting. The OTA International community would like to express its gratitude to all the authors who contributed their work in this volume.
